# An algorithmic approach to convex fair partitions of convex polygons

**DOI:** 10.1016/j.mex.2023.102530

**Published:** 2023-12-23

**Authors:** Mathilda Campillo, María D. González-Lima, Bernardo Uribe

**Affiliations:** University of the North, Barranquilla, Colombia

**Keywords:** Convex equipartition, Fair partition, Lloyd's algorithm, Voronoi partition, Centroidal Voronoi partition, Convex Fair Partitions Algorithm

## Abstract

A convex fair partition of a convex polygonal region is defined as a partition on which all regions are convex and have equal area and equal perimeter. In this article we describe an algorithm that finds such fair partition.

• The Fair Partitions method finds a fair partition for any given convex polygon and any given number of regions.
• Our method relies on two well-known methods: Lloyd's algorithm and the Normal Flow Algorithm.
• The method proposed in this article can be used in various contexts and many real-world applications.

## Graphical abstract

**Figure:**
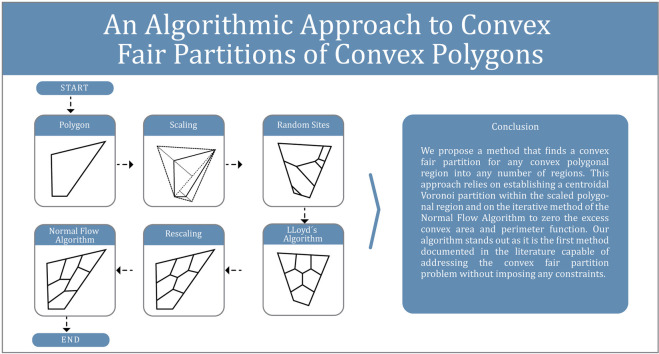

Specifications Table

**Table:** 

Subject area:	Mathematics and Statistics
More specific subject area:	Combinatorial Optimization
Name of your method:	Convex Fair Partitions Algorithm
Name and reference of original method:	N.A.
Resource availability:	https://github.com/Mathilda27

## Introduction

Problems regarding partitions of regions are common optimization problems as they arise from real world applications. Many authors have addressed the problem of partitioning convex regions with specific properties [1]. Among these, one that stands out is partitioning a convex polygon into convex regions of equal area and equal perimeter. This problem was firstly proposed by Nandakumar and Rao [2] where such partition was coined *convex fair partition*.

In [3,4] the question of showing the existence of such partition was addressed and it was shown that a convex fair partition always exists when the number of regions is a power of a prime number. The existence of the solution in the general case seems to have been resolved in [5], where an inductive argument based on the results [3,4] is presented.

While the aforementioned works tackle the issue of existence by using topological arguments that extend the famous Borsuk-Ulam Theorem, none of them address the practical challenge of finding such a partition for any given convex polygonal region and any given number of regions. Only when the number of regions is a power of 2, there exist an algorithm presented by Daescu [6] where the original idea of Nandakumar and Rao [2] is implemented.

In this work we explicitly describe the algorithm introduced in [7] that finds a solution to the problem of partitioning a convex polygon into convex regions of equal area and equal perimeter. Our protocol relies on two very well-known methods: *Lloyd's algorithm* that finds centroidal Voronoi partitions [8,9] and the generalization of Newton's method denoted *Normal Flow Algorithm* [10] that finds zeros of underdetermined systems of nonlinear functions.

An application to this problem is to consider a landmass with a convex border. In this case we want to divide the landmass into smaller regions that have the same area and same border. In fact, we can replace the functional of area and perimeter by any other that incorporates information obtained from the interior and the boundary, thereby solving many other similar problems. One such problem appears in electrical engineering. Consider a surface that is crossed by an electric current. The question of splitting the surface into a given number of panels such that the contour of each of them has the same electric current, and the rate of change of the magnetic field in time over each of them is the same, can be solved with a similar approach using the algorithm presented for the fair partition of convex polygons problem.

**Fig. 1:**
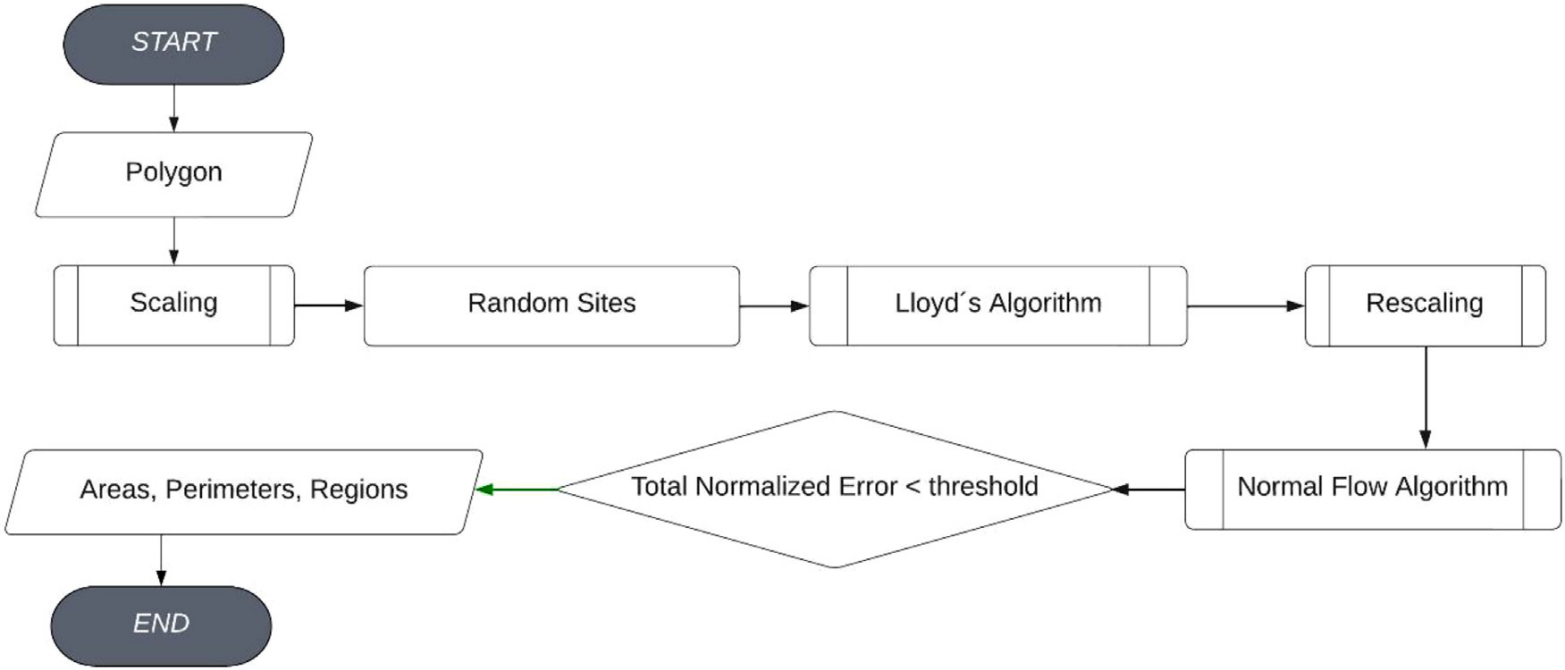
Flowchart of the fair partitions method.

Our algorithm stands out as the first method documented in the literature capable of addressing the convex fair partition problem without imposing any constraints (Fig. 1).

## Method details

The main algorithm we present in this work is the following (Algorithm 1):

**Algorithm 1:** Convex Fair Partition method (main algorithm).

**Input:** Convex polygon, number of partitions = *n*
**Output:** Convex fair partition in terms of areas, perimeters, and regions

1. Scale the height of the polygon along the direction perpendicular to its width to obtain a region whose height equals the original width. 2. Choose n random points in the interior of the scaled polygon. 3. Apply Lloyd's algorithm to find a centroidal Voronoi partition in the scaled polygon. 4. Scale back the centroidal Voronoi partition to the original polygon. 5. Apply the Normal Flow Algorithm to the excess convex area and perimeter functions.

**Fig. 2:**
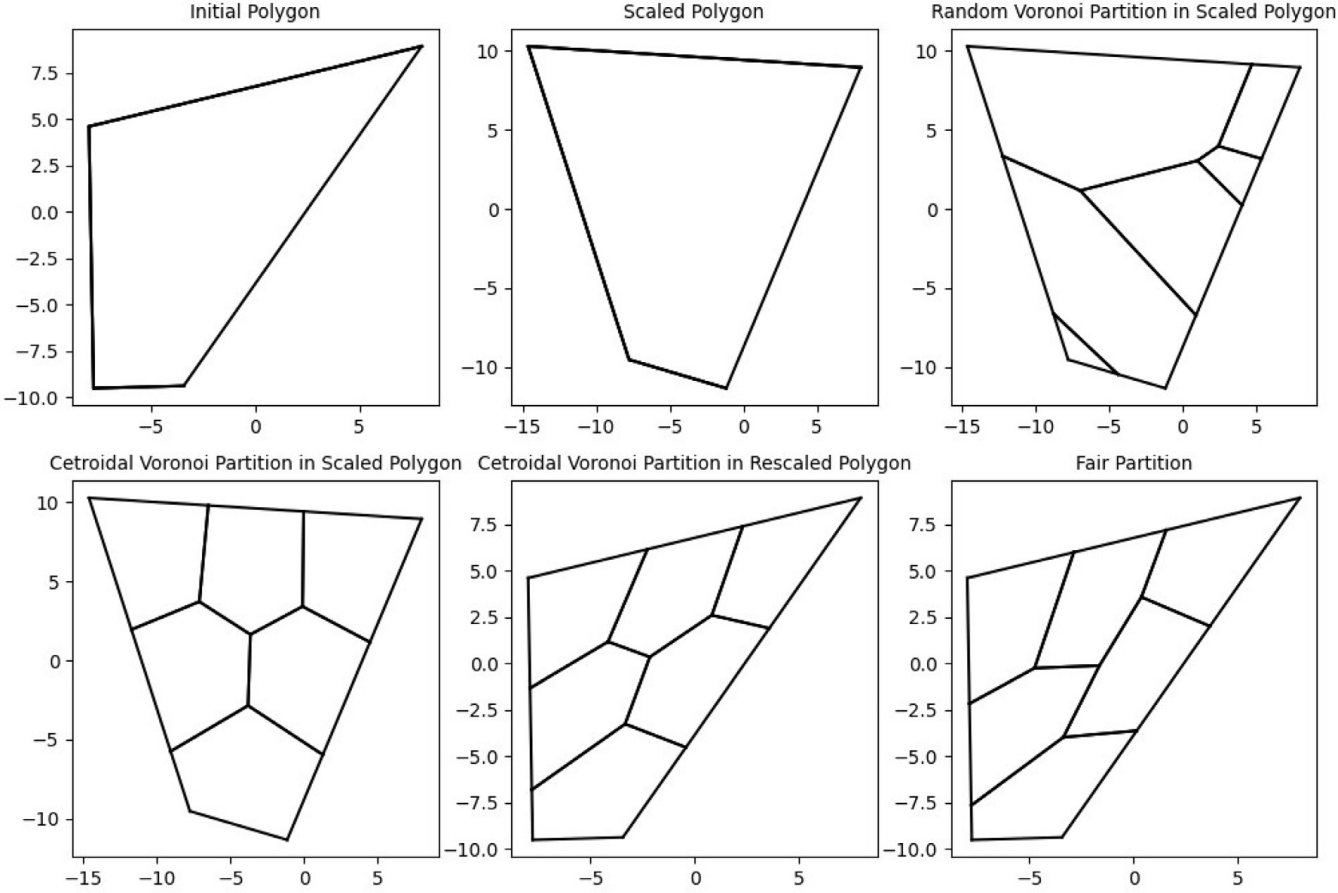
Output of the implemented protocol. This figure outlines the steps performed by our algorithm to find a convex fair partition of a random convex polygon.

In words, take a fixed convex polygon and construct a convex partition of the original polygon using Voronoi diagrams and centroidal Voronoi diagrams via Lloyd's Algorithm. Note that the vertices obtained by the resulting partition are either located in the interior, in the boundary, or are vertices of the polygon. The internal and boundary vertices, together with the edges of the regions that join them, define a combinatorial graph. By keeping the combinatorial configuration of the graph but perturbing the coordinates of the internal and boundary vertices, we are able to define a new geometrical partition of the original polygon. If the partition that we start with is located close to the solution, a generalized Newton's method such as the Normal Flow Algorithm can find the desired partition. This method is applied to the excess convex areas and the excess perimeters functions, the former one being the difference between the areas of the Convex Hulls (the smallest convex set that contains the region) and 1/*n*th of the area of the polygon, and the later one being the difference between the perimeters and their means. The zeroes of these functions provide a partition which is fair and moreover convex. See Fig. 2 to follow the implementation of our method.

## Voronoi partition & polygon intersections

**Fig. 3:**
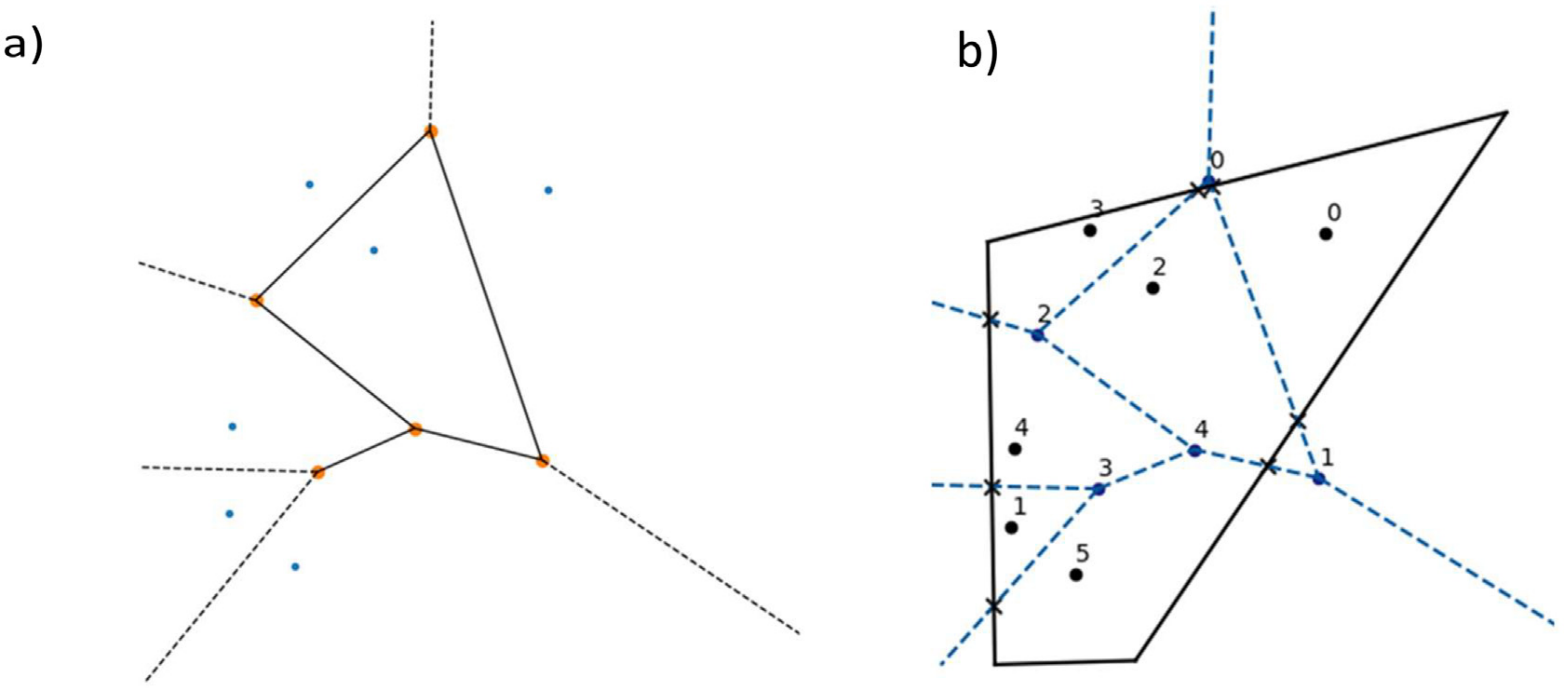
(a) Voronoi partition induced by 6 random Voronoi centers, vertices: coordinates of the vertices of the graph induced by the Voronoi partition, ridge_vertices: indices of the vertices of the graph induced by the Voronoi partition, ridge_points: indices of the Voronoi centers, black dotted lines represent unbounded rays. (b) Intersections between the Voronoi partition and the polygonal region. Here the Voronoi centers are numbered.

One of the most important steps of our method is to find the points of intersection between a fixed convex polygon and a fixed Voronoi partition. Voronoi partitions can be determined entirely by the coordinates of its centers and the combinatorial information of its points, edges and connections. Thus, by processing the data from the partition appropriately, we were able to find the intersections between the convex polygonal region and Voronoi partition. In order to achieve that, our Python implementation used the following information provided by scipy.spatial.Voronoi [11] as input for our script:

• ridge_vertices: Indices of the Voronoi vertices forming each Voronoi ridge, that is, the lines that bound each Voronoi cell.
• vertices: Coordinates of the Voronoi vertices, namely the vertices of the Voronoi cells.
• ridge_points: Indices of the Voronoi centers which are equidistant to each Voronoi ridge.
• regions: Indices of the Voronoi vertices forming each Voronoi region. An index of −1 indicates a vertex outside the Voronoi diagram.
• point_region: Index of the Voronoi region for each input Voronoi center (Fig. 3).

When calculating the intersections between the fixed polygonal region and the Voronoi partition induced by the input points, we noticed that many regions were unbounded, that is, at least one of its edges was an unbounded ray (contained a −1 index). To address this case, we propose Algorithm 2.

**Algorithm 2:** Intersections between a convex polygon and a Voronoi partition.

**Input:** Convex polygon, Voronoi centers.
**Output:** Partition

1. Use the Voronoi command from Scipy's Library to get the Voronoi diagram induced by the set of Voronoi centers. 2. Create a copy of the regions array, we will call it new regions. 3. Fix an index of the new regions array and delete any −1 index that may be inside it. 4. Delete from the new region's array the indices of the Voronoi vertices outside the polygonal region. 5. a) Append to the new region's array the vertices of the polygon which are closer to the Voronoi center of the region. b) If there is no −1 index in the region. Go over the lines that define the polygon and the lines that define the bounded region and find all the possible intersections. Append them to the new region's array. 6. If there is a −1 index in the region. Find the Voronoi centers that define the unbounded ridge and calculate its normal vector. The vector perpendicular to this normal vector determines the direction of the unbounded ridge. Find the closest Voronoi center to the vertex from which the unbounded ray starts. Determine the orientation of the unbounded ray and follow the previous step. 7. Sort the vertices in the new regions array in counterclockwise direction.
Calculate the perimeters and areas of the regions using the new regions array.
By following this algorithm, we were able to calculate the intersections of a Voronoi diagram with a polygon, that is, finding the Voronoi diagram defined by the input points bounded by the polygonal region.

## Scaling

The scaling of the polygon procedure goes as follows. Take the width of the polygon, namely the longest distance on the hull of the region. Measure the longest heights on each side of the width, add them up, and call this number the height of the polygon. Scale the polygon linearly on the direction perpendicular to the width such that in the scaled polygon the height equals the original width. This resulting polygon behaves better with respect to the centroidal Voronoi partition, in the sense that the number of internal points of the associated to the Voronoi partition is bigger than in the case of the original unscaled polygon.

**Fig. 4:**
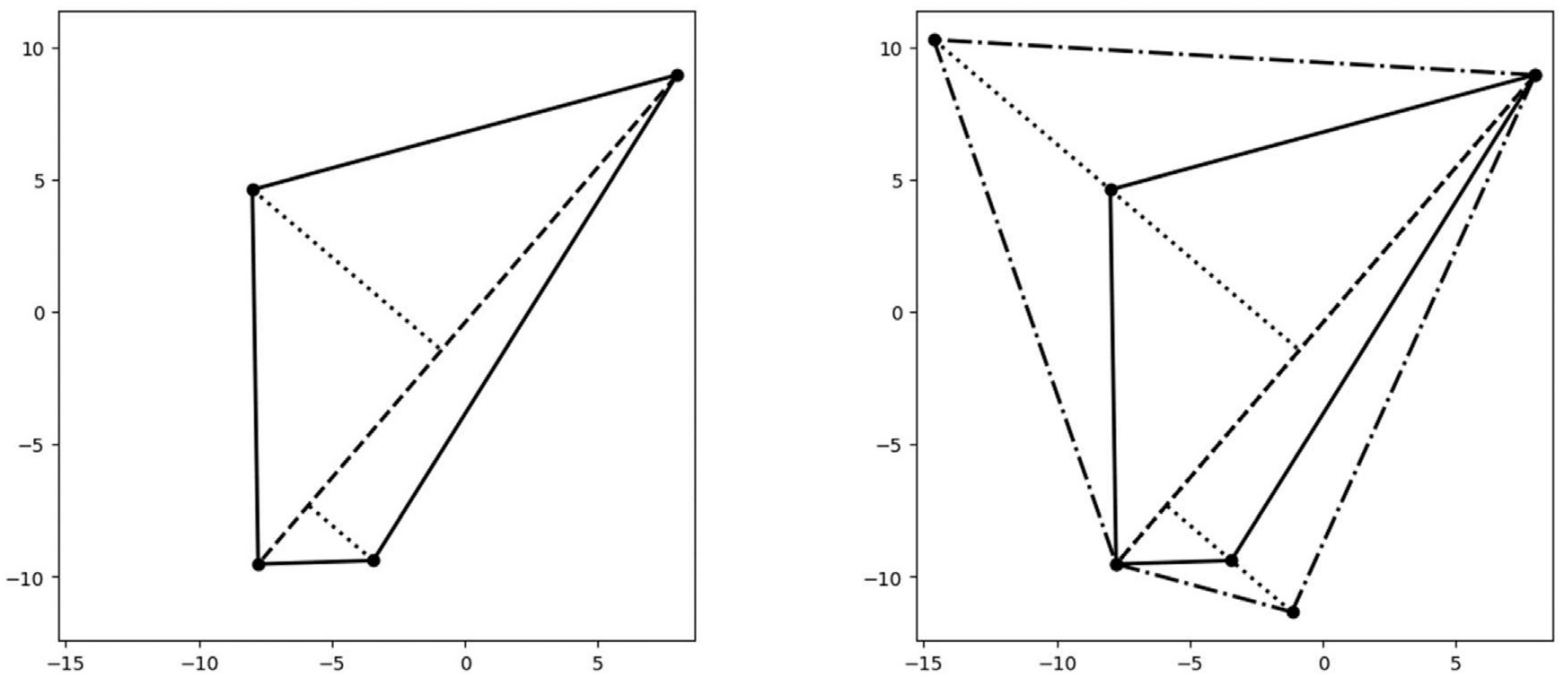
This figure describes the scaling protocol. The initial polygonal region is on the right side, and the scaled polygonal region on the left.

By applying this protocol we are able to obtain a centroidal Voronoi partition that has more internal vertices than the centroidal Voronoi partition of the original unscaled region. We noticed that the induced partition provides more room to maneuver for the Normal Flow Algorithm. Therefore, this protocol is necessary when facing some unbalanced or thin regions since our algorithm underperforms in the unscaled setup (Fig. 4).

## Lloyd's algorithm

Once obtained a balanced partition we need to determine a procedure that provides a starting convex partition close enough to a fair partition. In our case, Lloyd's algorithm is a very useful tool due to its simplicity. The original Lloyd's algorithm is presented below (Algorithm 3).

**Algorithm 3:** Lloyd's algorithm.

**Input:** Convex polygon, Voronoi centers.
**Output:** Centroidal Voronoi centers.

1. Compute the centroid of each Voronoi region.
2. Move each Voronoi center to its corresponding centroid.
3. Iterate until a threshold is achieved.

**Fig. 5:**
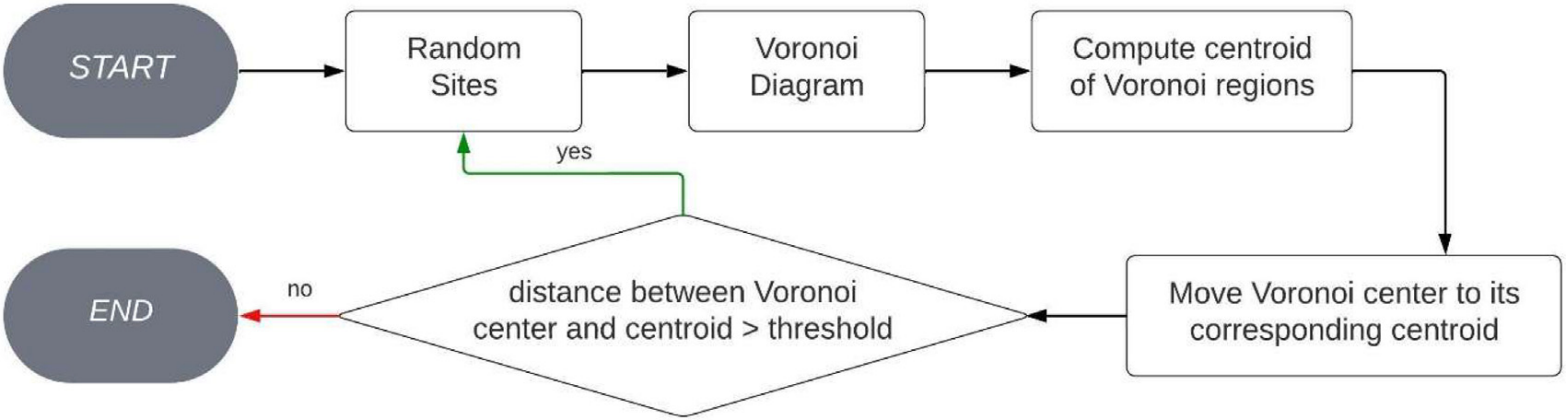
Original Lloyd's algorithm flowchart.

We start by applying Lloyd's algorithm to a random set of points inside a convex and bounded region. We then calculate the Voronoi partition that these points define and calculate the centroids of the regions the partition induces (Fig. 5).

**Fig. 6:**
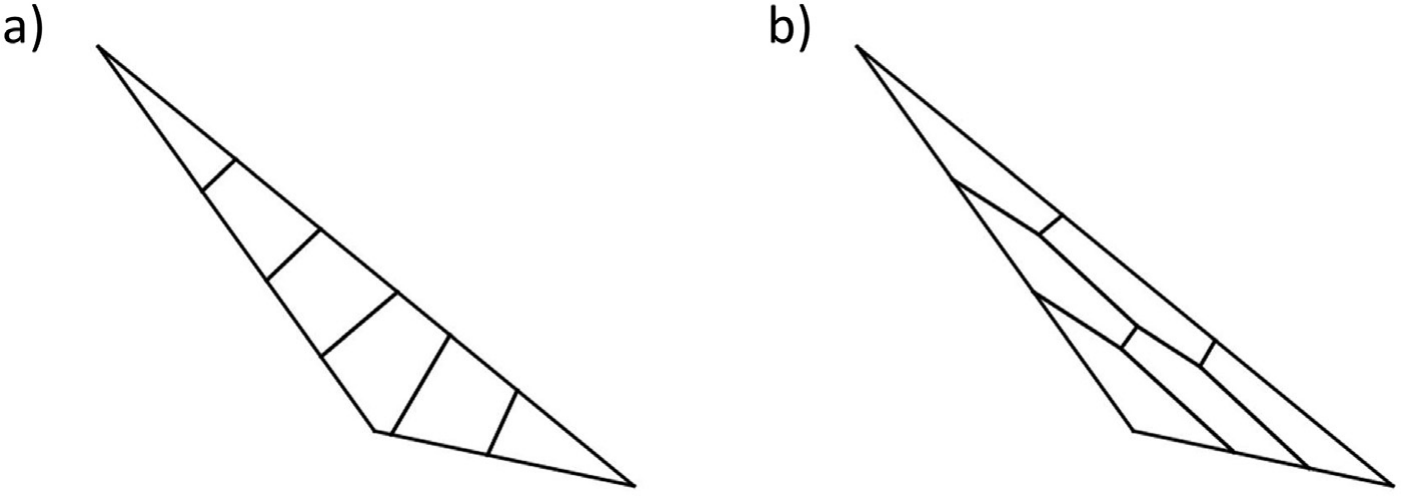
(a) Centroidal Voronoi partition performed on the original polygon, (b) Centroidal Voronoi partition performed on the scaled polygon. Note that the number of internal vertices is bigger on the scaled region.

By moving the Voronoi centers to their corresponding centroids, and iterating the procedure, Lloyd's algorithm converges to a centroidal Voronoi partition. These partitions provide subdivisions of the polygon which are very symmetrical with respect to the centroid. Additionally, the areas and perimeters of centroidal Voronoi partitions are similar, and therefore provide a decent starting partition for the Normal Flow Algorithm. We will later see that the number of internal vertices plays an important role in the convergence of the Normal Flow Algorithm. Lloyd's Algorithm applied to thin regions fails to provide an appropriate starting point as the number of internal vertices might be very small (compare the centroidal Voronoi partition of Fig. 6a with the scaled centroidal Voronoi partition of Fig. 6b).

Thus, we proceed to apply Lloyd's algorithm to a scaled polygon to find a starting partition that is close to the solution and has many internal vertices.

## Rescaling

To solve the original problem, that is, to find a fair partition of the initial polygon, we will have to reverse the scaling process. This is performed by the Rescaling function, which is very much the same as the Scaling function but changing the constant used for the linear scaling. This will not only give us a version of the centroidal Voronoi partition scaled to the original polygon but will also return the scaled polygon into its original shape. By applying this protocol, we make sure to obtain a partition with many internal vertices.

## Normal flow algorithm

Once obtained a suitable starting partition provided by the previous four steps, let us describe the nonlinear system problem we are facing. To obtain a proper set of equations we carried out the following steps:

1. Save the combinatorial information of the partition, this is, how the vertices and edges are connected to one another. Identify which vertices lie in the boundary and in the interior or are the vertices of the original polygon. This ensures that the initial structure of the partition is preserved under movements of the Voronoi vertices. This information will be used and kept fixed (this is essential to maintain the initial Voronoi partition configuration) during the whole process.
2. Calculate the convex area (area of the Convex Hull of the region) and perimeter of each region in terms of its vertices and edges.
3. Normalize the areas by subtracting 1/n-th of the area of the original polygon to each convex area and subtract the average of the perimeters to each region's perimeter. These are the convex excess area and perimeter functions.
4. Bundle up these excess area and perimeter functions into a functional *F* that parameterize the movements on the Voronoi vertices. Note that the total area of the original polygon is constant, and the total perimeter of the partition can be estimated, therefore we disregard the area and the perimeter of the last region, thus making *F* a functional with 2(n-1) outputs (n-1 excess convex areas and n-1 excess perimeters).

**Fig. 7:**
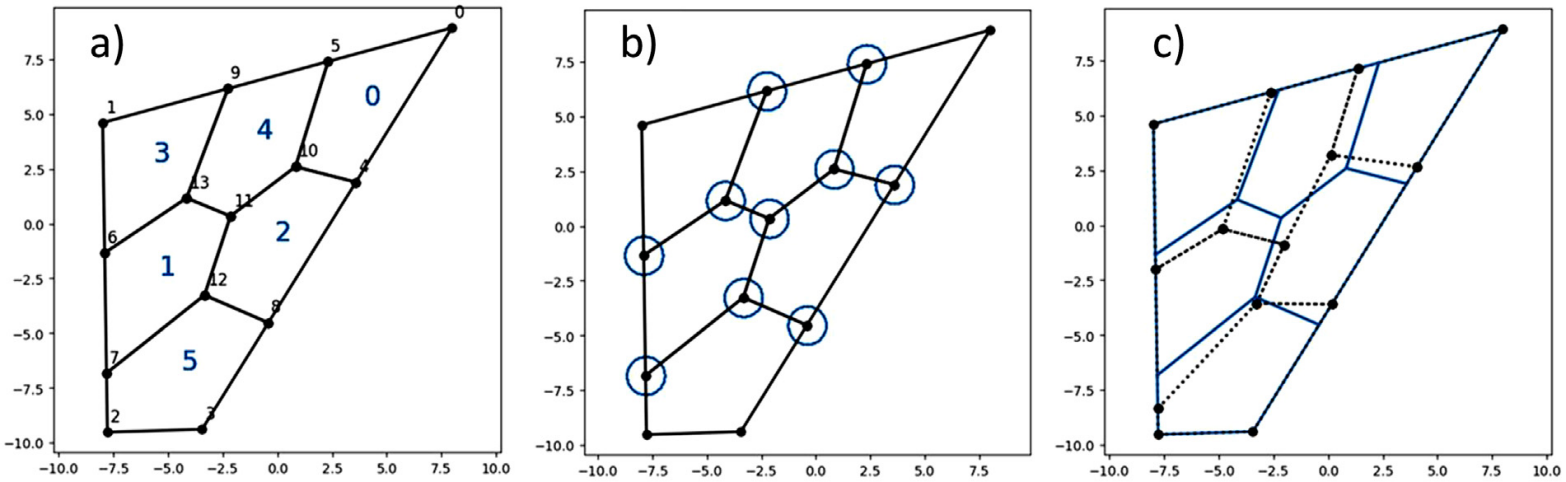
(a) Convex partition with numbered regions and vertices. In this case the indices of the external vertices are [0,1,2,3], of the boundary vertices are [4,5,6,7,8,9] and of the interior vertices are [10,12,13]. Neighborhoods of equal radius for the interior and boundary vertices. Moving the coordinates of the vertices inside those neighborhoods produces a new partition of the polygon with no overlapping of edges nor vertices. (c) The partition in broken lines is a perturbation of the original partition in solid lines.

The nonlinear system of equations can now be stated as follows: starting with a fix convex partition of a convex polygon, define the excess area and perimeter functional *F*, and find the solutions to the equation *F* = 0. A solution of *F* = 0 will provide the coordinates of a partition which is fair and moreover convex. Otherwise, the sum of the convex areas of all regions would be greater than the area of the polygon, implying that at least one of them is non-convex (Fig. 7).

One of the methods used for finding zeroes of functions is a generalization of Newton's algorithm called *Normal Flow Algorithm*. The *Normal Flow Algorithm* was first introduced by Ben-Israel [10] for solving nonlinear system of equations when the derivative matrix is not square. In this case we have a problem that has less equations than variables.

Through numerical experimentation, we noticed that the following modifications applied to the iteration step of the Normal Flow Algorithm applied to *F*, made the algorithm more effective in terms of finding the convex fair partition.

## Modifications

1. On the first iterations of the step of the Normal Flow Algorithm our protocol can become unstable. Therefore, after several trials we decided to shorten by $\frac{1}{10-i}$ the step of the first 10 iterations, that is, by $\frac{1}{10}$ on the first iteration, $\frac{1}{9}$ on the second, and so on, until the tenth iteration where subsequent steps are not modified.
2. The step is shortened by one half, whenever the iteration step sends any internal or boundary vertex outside the polygonal region.

The first procedure avoids that the first steps change dramatically the convexity, and the second prevents the internal and boundary vertices from abandon the polygonal region. Algorithm 4 describes our implementation of the Normal Flow Algorithm.

**Algorithm 4:** Normal flow algorithm.

**Input:** Internal vertices, boundary vertices, external vertices
**Output:** Areas, perimeters

1. Take the starting point for the Normal Flow algorithm as the value of F after the rescaling procedure. 2. Using numerical methods find the Jacobian matrix of F. 3. Find a solution to the system $F^{\prime }\left(x_{k}\right)d_{k}=-F\left(x_{k}\right)$ with minimal Euclidean norm. 4. Determine if an adjustment to the iteration step is required. If so, call the modified step $d_{k}^{\prime }$, otherwise $d_{k}=d_{k}^{\prime }$ 5. Move in the direction $d_{k}^{\prime }$, that is, the iteration step is $x_{k+1}=x_{k}+d_{k}^{\prime }$. 6. Iterate steps 2 to 5 until the threshold is achieved.

Note that the total dimension of the movements that can be carried out this way can be calculated using the Euler characteristic of the polygon and combinatorial properties of Voronoi diagrams. This total dimension is always greater or equal than the rank of the Jacobian matrix of F, and moreover, the extra dimensions equal the number of internal vertices, i.e*.* $dim(ker(F^{\prime }))=|I|$, where |I| denotes the number of internal vertices.

## Method validation

**Fig. 8:**
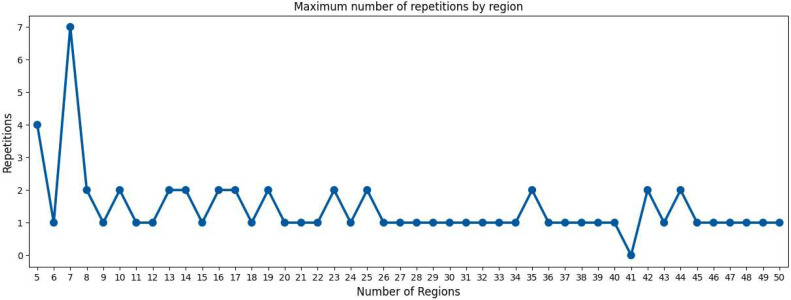
We have tested our Main Algorithm in 500 random convex polygons. For every number of regions between 5 and 50 we have plotted the maximum repetitions. 94.69 % of the regions were solved in one run, 99.93 % of the regions were solved in less than 3 runs, and 100 % of the regions were solved in at most 7 runs.

We have applied our main algorithm to 500 random convex polygons for each number of regions ranging between 5 and 50. The random polygon is obtained by taking the Convex Hull of 8 random points in the square [−10,10]×[−10,10] using a normal distribution on each variable. If our main algorithm did not find a fair partition, we restarted the protocol on the same polygonal region, up to 30 times. In order to have a similar metric to stop our Main Algorithm for different number of regions, we have measured the norm of the percentages of the excess in convex areas and perimeters and decided that our Main Algorithm succeeded whenever a value under $10^{-6}$ is achieved. This implies that all areas and all perimeters have a portion of excess of at most $10^{-6}$ times the mean area or the mean perimeter respectively. Our method finds a fair partition in at most 7 runs (Fig. 8).

**Fig. 9:**
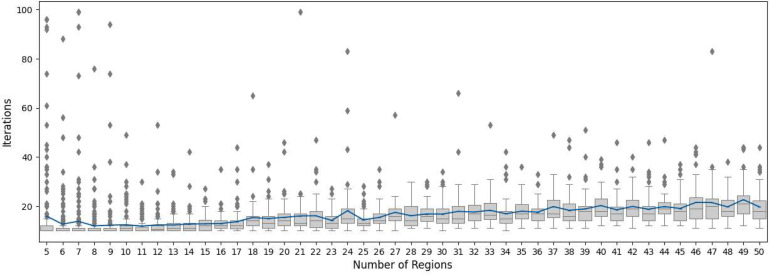
This box plot shows the number of iterations for every number of regions from 5 to 50 our Main Algorithm needed to find a convex fair partition. The line in blue is the mean number of iterations for each region.

This is strong evidence that our main algorithm indeed finds a convex fair partition for any convex polygonal region and any number of regions. However, it does have some limitations. In some cases, when the starting point is not suitable, the restart of the protocol with new initial Voronoi centers is required (Fig. 9). Additionally, the complexity of the Voronoi partition calculation is proportional to the number of regions, that is, $O(n^{2})$ where n is the number of regions.

To summarize, we have described a method that finds a convex fair partition for any convex polygonal region into any number of regions. This approach relies on establishing a centroidal Voronoi partition within the scaled polygonal region and on the iterative method of the Normal Flow Algorithm to zero the excess convex area and perimeter function. Our algorithm stands out as it is the first method documented in the literature capable of addressing the convex fair partition problem without imposing any constraints.

## Ethics statements

Our work did not require human subjects, animal experiments nor data collected from social media platforms.

## CRediT authorship contribution statement

**Mathilda Campillo:** Software, Validation, Investigation, Data curation, Visualization, Writing – review & editing, Writing – original draft. **María D. González-Lima:** Conceptualization, Methodology, Formal analysis, Writing – review & editing. **Bernardo Uribe:** Conceptualization, Methodology, Software, Validation, Formal analysis, Investigation, Data curation, Writing – review & editing, Visualization, Supervision.

## Declaration of Competing Interest

The authors declare that they have no known competing financial interests or personal relationships that could have appeared to influence the work reported in this paper.

## Data Availability

No data was used for the research described in the article.
